# Microstructural white matter alterations and hippocampal volumes are associated with cognitive deficits in craniopharyngioma

**DOI:** 10.1530/EJE-18-0081

**Published:** 2018-03-16

**Authors:** S Fjalldal, C Follin, D Svärd, L Rylander, S Gabery, Å Petersén, D van Westen, P C Sundgren, I M Björkman-Burtscher, J Lätt, B Ekman, A Johanson, E M Erfurth

**Affiliations:** 1 Department of EndocrinologySkåne University Hospital, Lund, Sweden; 2 Department of Diagnostic RadiologyClinical Sciences; 3 Division of Occupational and Environmental MedicineDepartment of Experimental Medical Science, Lund University, Lund, Sweden; 4 Translational Neuroendocrine Research UnitDepartment of Experimental Medical Science, Lund University, Lund, Sweden; 5 Department of Medical Imaging and PhysiologySkåne University Hospital, Lund, Sweden; 6 Department of Endocrinology and Medical and Health SciencesLinköping University, Linköping, Sweden; 7 Department of Psychology and PsychiatrySkåne University Hospital, Lund, Sweden

## Abstract

**Context:**

Patients with craniopharyngioma (CP) and hypothalamic lesions (HL) have cognitive deficits. Which neural pathways are affected is unknown.

**Objective:**

To determine whether there is a relationship between microstructural white matter (WM) alterations detected with diffusion tensor imaging (DTI) and cognition in adults with childhood-onset CP.

**Design:**

A cross-sectional study with a median follow-up time of 22 (6–49) years after operation.

**Setting:**

The South Medical Region of Sweden (2.5 million inhabitants).

**Participants:**

Included were 41 patients (24 women, ≥17 years) surgically treated for childhood-onset CP between 1958–2010 and 32 controls with similar age and gender distributions. HL was found in 23 patients.

**Main outcome measures:**

Subjects performed cognitive tests and magnetic resonance imaging, and images were analyzed using DTI of uncinate fasciculus, fornix, cingulum, hippocampus and hypothalamus as well as hippocampal volumetry.

**Results:**

Right uncinate fasciculus was significantly altered (*P* ≤ 0.01). Microstructural WM alterations in left ventral cingulum were significantly associated with worse performance in visual episodic memory, explaining approximately 50% of the variation. Alterations in dorsal cingulum were associated with worse performance in immediate, delayed recall and recognition, explaining 26–38% of the variation, and with visuospatial ability and executive function, explaining 19–29%. Patients who had smaller hippocampal volume had worse general knowledge (*P* = 0.028), and microstructural WM alterations in hippocampus were associated with a decline in general knowledge and episodic visual memory.

**Conclusions:**

A structure to function relationship is suggested between microstructural WM alterations in cingulum and in hippocampus with cognitive deficits in CP.

## Introduction

Craniopharyngioma (CP) is a benign pituitary tumor, however, known for its aggressive behavior, high recurrence rate (1) and increased mortality (2, 3, 4) and morbidity in cardiovascular diseases (5, 6). The cause is primarily due to hypothalamic lesion (HL) by the tumor or its treatment (6). The morbidity includes cognitive dysfunction with attention deficits, impaired episodic memory and processing speed (7, 8). Lately, focus has shifted toward data reflecting impairment in different cognitive domains and how HL contributes to a worse outcome (7, 9). The white matter (WM) of the brain is receiving increasingly importance in cognitive research (10) and diffusion tensor imaging (DTI) is a technique that allows noninvasive, *in vivo* study of the brain by assessing the motion of water molecules along and across neural axons (11, 12). Different DTI maps show the microstructural WM architecture (13) and can depict WM abnormalities. WM integrity is measured by fractional anisotropy (FA) and lower FA values are associated with decreased WM integrity (14). FA is sensitive to damage but is fairly nonspecific. The degree of directionality of water molecules is quantified by the DTI summary measure mean diffusivity (MD), which quantifies directionality, and increased MD may be caused by for example, demyelination or edema (14).

The prefrontal cortex is important for attention and executive function, a pre-requisite for working memory, short-term memory and a successive long-term memory. DTI in humans has shown direct connectivity between several hypothalamus (HT) compartments to WM areas including the frontal cortex (15). Animal studies have shown connectivity between the hippocampus and the HT (16) as well as directly to the frontal cortex (17). Thus, HL might indirectly lead to frontal and medial temporal lobe dysfunction causing deficits in memory, attention and executive function (18). In children with CP, the consequences of HL have been investigated with functional magnetic resonance imaging (fMRI) showing a defect in neural correlates of memory retrieval in the medial prefrontal cortex (19). Further, pre-existing surgical defects after CP operation appeared to accentuate the radiation dose effect on WM (20). Few studies have investigated the HT using DTI in human subjects (21, 22, 23), but in obese subjects, HT microstructural alterations were associated with worse cognitive performance (24).

The hippocampus is important for encoding and retrieving sequences of events that compose particularly the episodic (contemporary) memory (25). The cingulum is an important WM tract with multiple reciprocal connections with the hippocampus and well-known importance for episodic memory (26, 27). The uncinate fasciculus is also important for episodic memory and connects limbic regions in the temporal lobe to the frontal lobe (28) and the fornix connects the hippocampus to the prefrontal cortex (29).

The aim of this study was to investigate whether there is a structure to function relationship between WM alterations detected with DTI and cognition in adults with childhood-onset CP. We analyzed DTI in neural pathways of importance and also DTI in the HT and hippocampus, as well as hippocampal volumetry.

## Methods

### Patients

Forty-one (24 women) patients, aged ≥17 years, with a median age at investigation of 35 (range 17–56) years in women, and 36 (20–49) years in men, were recruited from 64 eligible subjects from the Southern region of Sweden (population 2.5 million). The patients were surgically treated for a childhood-onset CP between 1958 and 2010 at Skåne University Hospital. Excluded subjects (*n* = 23) were either assessed to be too ill (meningioma *n* = 1, neuromuscular disease *n* = 1, living in a home for disabled *n* = 2), too busy (*n* = 6), investigations to be stressful according to patients (*n* = 2), had aneurysm clip (*n* = 1), did not give any reason (*n* = 7), had missing medical records (*n* = 1) or did not reply (*n* = 2).

Five patients had to withdraw from MRI due to presence of either a shunt causing significant MR artifacts (*n* = 1), pacemaker (*n* = 1), claustrophobia (*n* = 2) or weight restrictions (*n* = 1). These five patients were nevertheless included in the investigation of cognitive function (*n* = 41). A total of 36 patients completed MRI. Two patients were excluded from the hippocampal volume analysis after manual inspection revealed a discrepancy in the automated anatomical delineation of the hippocampus attributed to postoperative anatomical changes (*n* = 34). Four patients were excluded from the DTI data analyses due to technical problems (shunt, *n* = 2; silver clips, *n* = 1; causing insufficient resolution for DTI processing; *n* = 1). One further patient was excluded from the data analysis involving only the fornix and the HT as the imaging data did not fulfill the quality criteria for placement of the region of interest (ROI). Six additional patients were excluded from the DTI analyses of the HT as the extent of HL prevented the imaging data to fulfill the quality criteria for ROI placement. This resulted in a total of 32 patients completing the DTI part of the MRI analysis with the exception of fornix (*n* = 31) and HT (*n* = 25).

Patient baseline characteristics and tumor treatment modalities are shown in Table 1. Sixteen patients had received cranial radiotherapy (CRT), median dose 50 (35–55) Gy. Age at first operation was 12 (3–29) years in women and 9 (3–22) years in men and time since first operation was 21 (6–49) years in women and 23 (11–42) years in men. At the time of this study, the same neurosurgeon graded the tumor location retrospectively based on each patient’s operation records: intra-sellar growth, supra-sellar growth, supra-sellar growth toward or into the 3rd ventricle. The latter was a criterion for HL. Twenty-three patients had HL. At the time of the study 76% of patients received GH therapy. Median daily GH dose was 0.6 (0.4–1.2) mg in women and 0.5 (0.2–0.8) mg in men resulting in a normalization of serum insulin-like growth factor-I (IGF-I) in all patients. Seventy-one percent of the women were on oral sex steroid treatment and 1 woman also had androgen supplementation. The remaining women had normal gonadal function according to blood tests. Among men, 82% needed testosterone replacement. Eighty-three percent of the women and 88% of men received levothyroxine in doses of 150 (50–250) μg and 150 (100–200) μg, respectively, with s-free T4 values in women of 18 (11–29) pmol/L and men 17 (13–21) pmol/L (reference range 12–22 pmol/L). Five patients had normal adrenocorticotropic-cortisol axes and all others needed hydrocortisone in doses of 25 (20–40) mg in women and 20 (10–30) mg in men. None of the patients were smokers. Twenty-five patients had normal visual acuity (≥0.5) along with normal or minor visual field defects. Details are shown in Supplementary Table 1 (see section on supplementary data given at the end of this article).

**Table 1:** Patients’ baseline characteristics and tumor treatment modalities shown separate for patients with hypothalamic (HT) lesion and without.^*,≠^

Gender	Age at investigation (y)	Age at first operation (y)	Treatment	Hormone substitution
With HT lesion (*n* = 23)				
F	38	20	S	G/T^a^
F	46	27	S	GH^c^
F	36	12	S	GH/G/T/C/ADH^c^
F	56	7	S+CRT^∞^	GH/G/T/C
F	33	3	S+CRT+In	GH/G/T/C/ADH
F	33	5	S+CRT	GH/G/T/C/ADH
F	32	15	S+CRT	GH/G/T/C/ADH
F	29	13	S	GH/G/T/C/ADH
F	28	4	S+CRT	GH/G/T/C/ADH
F	40	22	S+In	GH/G/T/C/ADH
F	35	29	S	GH/G/T/C/ADH
F	19	7	S	GH/G/T/C/ADH
F	37	9	S+CRT	GH/G/T/C/ADH
M	43	9	S+CRT+In	GH/G/T/C/ADH^c^
M	20	9	S+CRT	None^b^
M	33	22	S+CRT	GH/G/T/C
M	49	12	S	GH/G/T/C/ADH
M	35	6	S+CRT+In+SR	GH/G/T/C/ADH
M	21	9	S	GH/G/T/C/ADH
M	38	16	S+CRT	GH/G/T/C/ADH
M	37	8	S+CRT	GH/G/T/C/ADH
M	35	16	S+CRT	GH/G/T/C/ADH
Without HT lesion (*n* = 18)				
F	49	12	S	ADH^a^
F	47	12	S	T/ADH^a^
F	25	21	S	T/ADH^b^
F	41	11	S	None^b^
F	34	15	S	GH/G/ADH
F	40	11	S	GH/G/T/C/ADH
F	38	9	S	GH/G/T/C/ADH
F	32	10	S	GH/G/T/C/ADH
F	29	17	S	GH/G/T/C/ADH
F	18	6	S+In+SR	GH/G/T/C/ADH
F	30	5	S+CRT	GH/G/T/C/ADH
M	40	17	S	ADH^a^
M	35	14	S	GH/G/T/ADH
M	47	5	S	GH/G/T/C/ADH
M	46	14	S	GH/G/T/C/ADH
M	37	3	S	GH/G/T/C/ADH
M	36	14	S	GH/G/T/C/ADH
M	27	4	S+CRT	GH/G/T/C/ADH

^*^Hypothalamic lesion according to the neurosurgeon’s retrospective assignment of patients to the non-hypothalamic lesion and hypothalamic lesion group based on operation records. ^≠^27 patients had 1 operation (13 hypothalamic lesion), 11 patients had 2 operations (8 hypothalamic lesion), 3 patients had 3 operations (2 hypothalamic lesion). ^∞^35 Gy Cobalt three-field’s technique. ^a^Intact GH axis based on insulin tolerance test. ^b^Intact GH axis based on clinical judgment. ^c^GH deficient but stopped GH treatment.

ADH, antidiuretic hormone; C, cortisone; CRT, cranial radiotherapy; F, female; G, gonadal steroids; GH, growth hormone; In, installation of yttrium; M, male; S, surgery; SR, stereotactic radiosurgery; T, levothyroxine; y, years.

### Control subjects

A control group consisting of 32 subjects (18 women) with similar age, gender and smoking habit distributions was established. Twenty controls were recruited from a pool of healthy subjects who were randomly selected and participated in our previous studies (7, 23). Twelve new controls were randomly selected from a computerized population register as previously described (30). One control subject terminated MRI due to claustrophobia and another was excluded from DTI involving hypothalamus (*n* = 31) based on quality criteria.

### Study design

The present investigations were performed during a single day in each patient.

The study was approved by the ethics committee (DNR 2011/769). All participants gave written informed consent.

### Cognitive tests

All 41 CP patients and 32 controls underwent neuropsychological assessment (Table 2) administered by two psychologists. WAIS vocabulary (Wechsler Adult Intelligence Scale) is a test of verbal knowledge representing semantic memory (31). WAIS digit span is a test of working memory, the subtest digit span backward measuring executive function. Rey Auditory Verbal Learning Test (RAVLT) was used to evaluate episodic verbal memory including immediate (1–2 min) and delayed recall (30 min) and recognition (31). Episodic visual memory was tested in a similar manner with Rey Complex Figure Test (31). Visuospatial abilities were tested with WAIS Block Design Test and the copy subtest from the Rey Complex Figure Test. Executive function, attention and processing speed were measured with WAIS coding and WAIS block test along with Trail making test.

**Table 2:** Neuropsychological test scores of 23^*^ childhood-onset CP patients with Hypothalamic (HT) lesion and 32 controls.

	Patients with HT lesion (*n* = 23)		Controls (*n* = 32)		*P*-value
	Median	10th–90th percentiles	Median	10th–90th percentiles	
Semantic memory (WAIS vocabulary)	31	18–40	38	30–47	**0.004**
Episodic memory					
Verbal memory (RAVLT)					
Immediate recall	51	31–65	57	44–68	0.054
Delayed recall	11	2–15	12	7–15	**0.021**
Recognition	13	10–15	15	12–15	**0.012**
Visual memory (Rey Complex Figure)					
Immediate recall	17	6–29	23	15–28	0.061
Delayed recall	17	6–27	23	13–28	**0.033**
Recognition	21	20–23	22	19–23	ns
Working memory (WAIS digit span)	27	19–33	27	19–37	ns
Executive function, processing speed					
WAIS digit span – backward subtest	9	5–12	9	6–13	ns
WAIS coding	71	50–88	75	59–101	ns
Trail making test					
Test 1	22	17–39	19	14–27	**0.025**
Test 2	25	19–39	22	16–32	ns
Test 3	28	22–41	23	15–37	**0.042**
Test 4	65	46–105	60	38–98	ns
Test 5	32	17–50	22	14–32	**0.001**
Visuospatial abilities					
Rey complex figure copy	35	32–36	35	32–36	ns
WAIS block design	52	30–60	52	31–60	ns

*n = 23 with following exceptions: WAIS block design *n* = 22 as one patient was excluded due to visual problems, WAIS coding *n* = 16 as 7 patients were excluded due to visual problems, Trail making test *n* = 16 as 7 patients were excluded due to visual problems, Rey Complex Figure Test *n* = 16 as 7 patients were excluded due to visual problems.

### Neuroimaging protocols

#### Data acquisition, post-processing and definition of FA and MD

Imaging sequences were acquired on a 3-Tesla MRI scanner (MAGNETOM Skyra, Siemens healthcare, Erlangen, Germany) using a 20-channel head/neck receiver coil. DTI data consisted of three volumes acquired with *b* = 0 s/mm^2^, followed by 96 volumes acquired using *b*-values of 250, 500, 1000 and 2750 s/mm^2^ distributed over 6, 6, 20 and 64 directions, respectively. In all 52 contiguous axial slices with a spatial resolution of 2.3 × 2.3 × 2.3 mm^3^ were acquired using a single-shot EPI (TR/TE 8100/103 ms/ms) sequence. For volumetric measurements, axial T1-weighted MPRAGE images were acquired (1 mm isotropic resolution, TE 3 ms, TR 1900 ms, flip angle 9). Motion and eddy current-induced artifacts were corrected using ElastiX (32). For each subject, DTI parameter maps for MD and FA were calculated using in-house developed software, implemented in Matlab (MATLAB 2013a, The MathWorks Inc., Natick, MA, USA). DTI volumes were registered to MNI152 standard space and to T1-weighted MPRAGE volumes using the registration algorithm of FLIRT and FNIRT (part of the FMRIB Software Library); (33) and ElastiX, respectively. The resolution of the resulting parameter maps was 1 × 1 × 1 mm^3^.

When all diffusion is parallel FA is near 1. Thus, WM alterations will result in lower FA and higher MD (12).

#### Tractography

**Figure 1:**
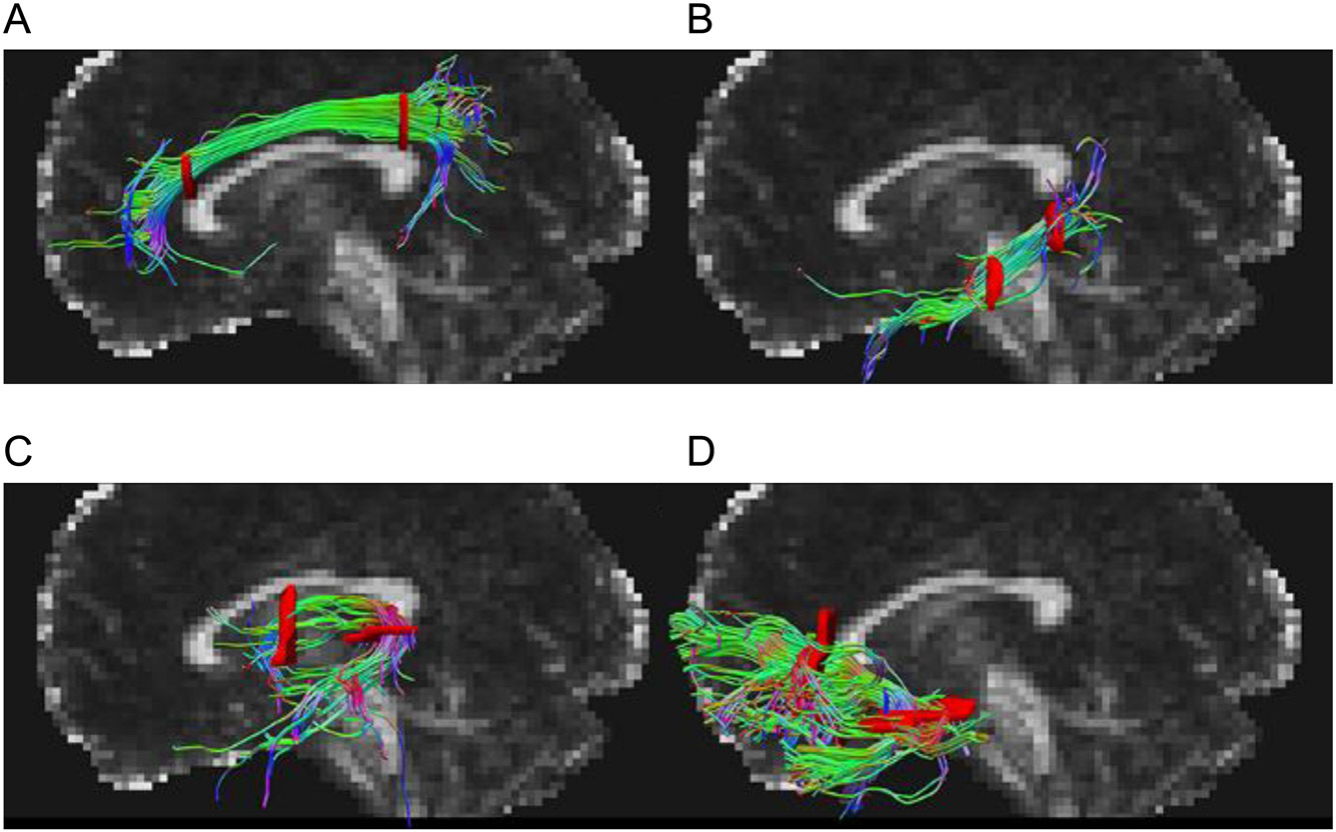
Graphical renderings of directionally color-coded (red, right-left; green, anterior-posterior; blue, superior-inferior) segmented tractography of (A) dorsal cingulum, (B) ventral cingulum, (C) fornix, and (D) uncinate fasciculus superimposed on a mid-sagittal FA map in a representative subject.

Tractography was performed using deterministic tracking based on constrained spherical deconvolution and generated the investigated white matter structures: the right and left dorsal cingulum, the right and left ventral cingulum, the fornix and the right and left uncinate fasciculus (Fig. 1). ROIs were used to define the seed region for each tract and to segment the tract based on Boolean operations. All ROIs were defined in MNI152 standard space and warped back utilizing the warp-fields generated by FNIRT to native space. All tracts were generated using one seed ROI covering the entire tract. The *dorsal cingulum* was defined using two ‘AND’ ROIs around the tract superior to the genu and splenium, respectively, and one ‘NOT’ ROI across one mid-sagittal slice. The *ventral (hippocampal) cingulum* was defined using two ‘AND’ ROIs around the tract rostral to the pons and inferior to the splenium, respectively, and one ‘NOT’ ROI across one mid-sagittal slice. The *fornix* was defined using two ‘AND’ ROIs around the body of the fornix caudal to the anterior pillars and around the crus fornici inferior to the splenium, respectively, and three ‘NOT’ ROIs rostral to the anterior pillars, caudal to the crus fornici and through the corpus callosum on axial slices, respectively. The *uncinate fasciculus* was defined using two ‘AND’ ROIs around the tract rostral to the genu and around the tract where it bends into the temporal lobe ventral to the upper pons, respectively, and three ‘NOT’ ROIs caudal to the front of the pons, through the corpus callosum on axial slices, and across one mid-sagittal slice, respectively. If the total number of streamlines generated for each WM tract was <100, the ROIs utilized for definition of the specific tract were visually inspected and adjusted if not located in the intended anatomical region. The average parameter estimates for each WM tract were used in the subsequent analysis.

#### DTI of hippocampus and HT

FreeSurfer was used to automatically segment hippocampus on T1-weighted volumes in native space (34). These segmentations were visually inspected and used as ROIs for delineation of the hippocampus on co-registered diffusion parameter maps to extract the mean diffusion parameter values from the right and left hippocampus. A ROI was manually defined on T1-weighted volumes in the right and the left HT according to the same HT delineation protocol as described in Gabery *et al*. (35). These ROIs were used to calculate the mean DTI parameter value from the co-registered DTI parameter maps and used in the subsequent analyses.

#### Volumetry of hippocampus

**Figure 2:**
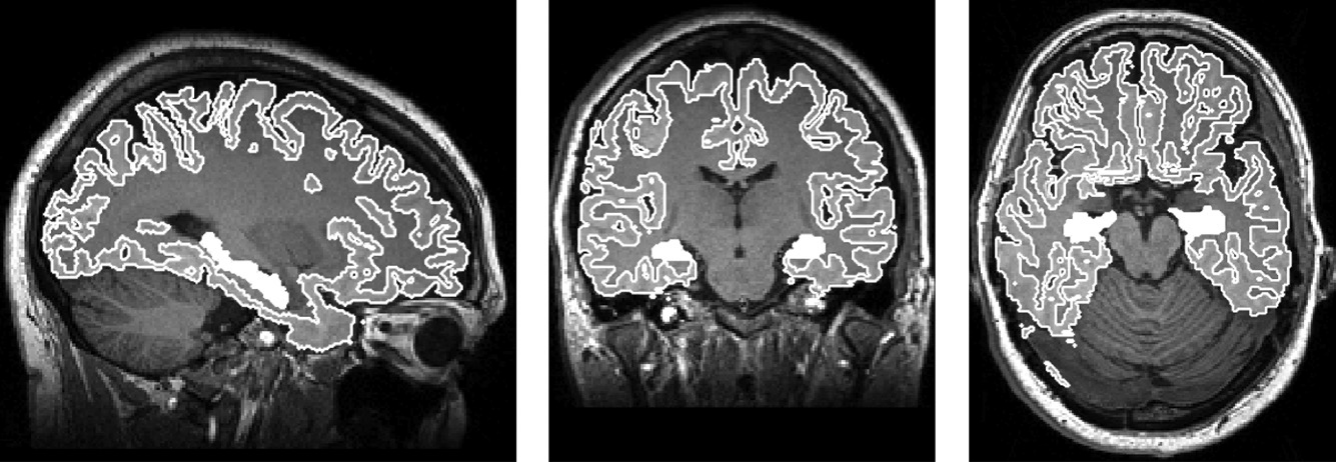
An example of a typical MPRAGE image in the three orthogonal planes, with Hippocampus region outlined in white, obtained from FreeSurfer (http://surfer.nmr.mgh.harvard.edu/).

The volumetric analysis was performed on the T1-MPRAGE images, using the FreeSurfer software package (version 5.1.0), freely available at http://surfer.nmr.mgh.harvard.edu/) (Fig. 2).

### Statistics

Patients’ characteristics are presented as median (min–max). Data on neuropsychological testing is presented as median and 10th–90th percentiles. Differences between patients and controls were compared using Mann–Whitney *U* test. Among the patients bivariate correlations were assessed using Pearson’s correlation coefficient (*r*). To ensure that the assumption of linearity was reasonable, scatter plots were investigated. Linear regressions were performed to investigate whether the estimates were confounded by age at investigation and intracranial volume (ICV). From the linear regression models, we presented the proportion of the variance explained by the variables included in the models (*r* ^2^). To ensure that model assumptions for the linear regressions were reasonably fulfilled, we investigated scatter plots as well as residual analyses. Only the statistically significant (defined as *P* < 0.05) results are presented. We used SPSS, version 22.0, for the statistical analysis.

## Results

### Cognitive comparison between CP patients and controls

No significant differences were recorded in any of the cognitive tests between the whole patient group (*n* = 41) and the controls (*n* = 32).

Patients with HL (*n* = 23) scored worse than the control group (*n* = 32) in the following tests (Table 2). A significant difference was found in semantic memory between patients and controls. In episodic memory, a significant difference in verbal memory, including delayed recall and recognition, was found between patients and controls. Further, a significant difference in episodic visual memory was found, on delayed recall. In executive function and processing speed, the Trail Making subtest 1, 3 and 5 measuring motor speed was significantly different between patients and controls.

### Comparison of DTI measures in CP patients and controls

Patients (*n* = 32) had higher MD and lower FA values only in right uncinate fasciculus compared to controls (*n* = 31) (*P* < 0.001, *P* = 0.01, respectively). No significant differences were found in DTI parameters in the HT (*n* = 25), hippocampus, cingulum or fornix when comparing patients and controls (Supplementary Table 2).

Patients with HL (*n* = 15) had higher MD and lower FA values in right uncinate fasciculus compared to controls (*n* = 31) (*P* = 0.005 and *P* = 0.011, respectively). No significant differences were found in DTI parameters in the HT (*n* = 8), hippocampus, cingulum or fornix, when comparing patients and controls (Supplementary Table 2).

### Associations between DTI measures and cognitive function

After adjustment for age at investigation and ICV among patients, an increase in MD of right dorsal cingulum was significantly associated with a decrease in episodic visual memory (Rey complex figure) for immediate recall (MD: *r* ^2^ = 0.29). This means that the model including dorsal cingulum (right), age and ICV explained 29% of the variance of Rey complex figure, immediate recall (MD: *r* ^2^ = 0.29, *P* = 0.02). Also a decrease in FA of this tract correlated with a decline in immediate recall (FA: *r* ^2^ = 0.38, *P* = 0.003). Comparable results were found for delayed recall (MD: *r* ^2^ = 0.26, *P* = 0.02 and FA: *r* ^2^ = 0.34, *P* = 0.004) and recognition (MD: *r* ^2^ = 0.32, *P* = 0.01). Further, also a decrease in FA in right dorsal cingulum correlated with a decline in visuospatial abilities (WAIS block design) (FA: *r* ^2^ = 0.29, *P* = 0.01) and an increase in MD correlated with a decline in executive function and processing speed (WAIS digit span backward) (MD: *r* ^2^ = 0.19, *P* = 0.01).

**Figure 3:**
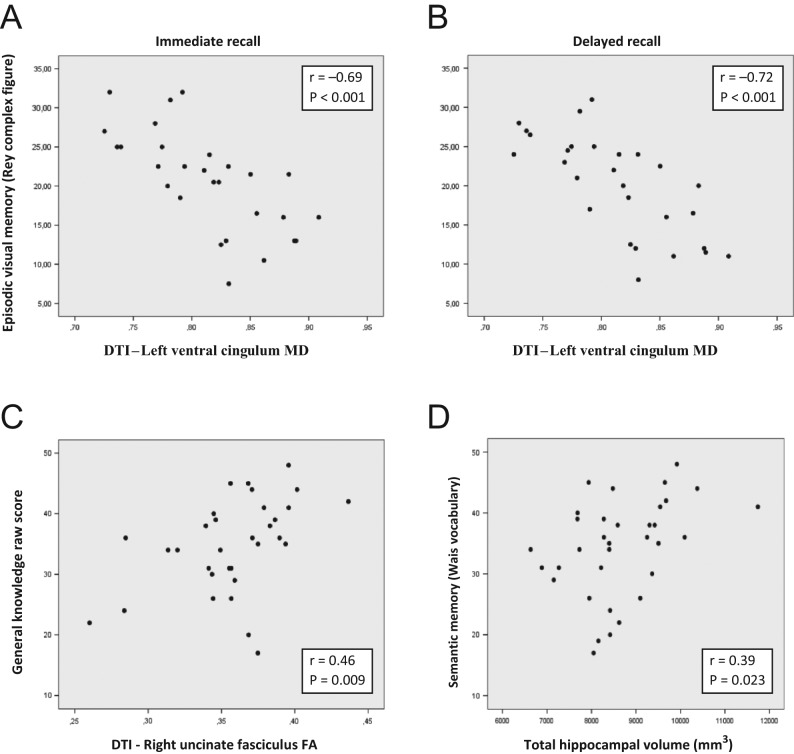
Episodic visual memory (Rey Complex Figure Test) with Immediate recall (A) and Delayed recall (B) in association to DTI in the left ventral cingulum (MD; mean diffusivity) among 32 CP patients. (C) Cognitive test of general knowledge in relation to DTI (FA; fractional anisotropy in the right uncinate fasciculus). Pearson’s correlation coefficient (*r*). (D) Cognitive test of General knowledge in relation to total hippocampal volume among 34 CP patients. Pearson’s correlation coefficient (*r*).

An increase in MD of the right ventral cingulum associated significantly with reduced episodic visual memory (Rey complex figure) for immediate recall (*r* ^2^ = 0.27, *P* = 0.03), delayed recall (*r* ^2^ = 0.29, *P* = 0.01) and recognition (*r* ^2^ = 0.31, *P* = 0.01). Further, an increase in MD of left ventral cingulum associated significantly with a reduced episodic visual memory for immediate recall (*r* ^2^ = 0.48, *P* < 0.001) (Fig. 3A), delayed recall (*r* ^2^ = 0.51, *P* < 0.001) (Fig. 3B) and recognition (*r* ^2^ = 0.43, *P* = 0.001).

A decrease in FA of the right uncinate fasciculus associated significantly with a decrease in semantic memory (WAIS vocabulary) (FA: *r* ^2^ = 0.19, *P* = 0.02) (Fig. 3C).

No associations were found involving the fornix and no associations between the above DTI measures and cognitive tests were recorded among the controls.

### Comparison of hippocampal volumes between all patients and controls and associations between hippocampal volumes and cognitive function among patients

After adjustment for age and ICV, the CP patients (*n* = 34) had smaller left and right hippocampal volumes compared to controls (*n* = 31) (4143 (3252–5852) mm^3^ vs 4478 (3570–5650) mm^3^ (*P* = 0.05) and 4290 (3378–5890) mm^3^ vs 4579 (3821–5927) mm^3^ (*P* = 0.02), respectively). The variation of hippocampal volumes in the study population (patients and controls) was largely explained by ICV, explaining 34% of the variance, while the group variable explained less than 5% of the variance.

Among CP patient (*n* = 34) hippocampal volumes, right and left side as well as total hippocampal volume, were significantly correlated with semantic memory (WAIS vocabulary) ((*r* = 0.38, *P* = 0.025), (*r* = 0.38, *P* = 0.027) and (*r* = 0.39, *P* = 0.023), respectively). After adjustment for age, the association between total hippocampal volume and semantic memory continued to be significant (*r* ^2^ = 0.12, *P* = 0.026) (Fig. 3D). No such associations were found among the controls.

### Associations between DTI in the hippocampus and cognitive function among patients

After adjustment for age (*n* = 32), a decrease in FA of the right hippocampus was significantly associated with reduced visuospatial abilities (WAIS block design) (FA: *r* ^2^ = 0.12, *P* = 0.04). An increase in MD and a decrease in FA of the left hippocampus associated significantly with reduced semantic memory (WAIS vocabulary) (MD: *r* ^2^ = 0.19, *P* = 0.01 and FA: *r* ^2^ = 0.26, *P* = 0.002) and episodic visual memory (Rey complex figure) for delayed recall (MD: *r* ^2^ = 0.13, *P* = 0.03 and FA: *r* ^2^ = 0.12, *P* = 0.04). No such associations were found among the controls.

## Discussion

In the present study, we show for the first time that late complications after CP treatment involve cognitive deficits that are associated with microstructural WM alterations detected with DTI in several neural pathways. Compared to controls, demyelination (increased MD) and loss of WM integrity (reduced FA) was recorded in the right uncinate fasciculus, and loss of WM integrity was associated with lower performance in general knowledge. Further, demyelination or edema of the cingulum (increased MD) was associated with a decline in episodic visual memory, visuospatial ability, executive function and processing speed. In addition, the patients have a smaller hippocampal volume associated with lower performance in general knowledge.

Our results show that CP patients have microstructural WM alterations (both reduced FA and increased MD) in the right uncinate fasciculus, a tract known to be important for general knowledge (28). In addition, reduced FA in this tract was associated with worse general knowledge. Importantly, no association was found among the controls. We have previously shown that CP patients tend to have a lower level of education (7). Although that could explain worse general knowledge, we argue that this may be a consequens of the disease, as uncinate fasciculus is among few neural pathways, which continue to mature until the early thirties (36), making it vulnerable to early treatment-related WM alterations. Also, in patients with Cushing syndrome, DTI has shown reduced WM integrity in the uncinate fasciculus (reduced FA), which was associated with a severity in depressive symptoms (37).

The ventral cingulum is highly connected to the medial temporal lobe important for episodic memory (38) and the strongest association was found between left ventral cingulum (increased MD) and worse episodic visual memory, for immediate recall (Fig. 3A), delayed recall (Fig. 3B) and recognition, explaining between 43 and 51% of the variation. Thus, the left ventral cingulum seemed to be particularly affected with demyelination or edema (14). This is in line with a prior study among patients with mild cognitive impairment and Alzheimer disease where early degeneration in the ventral cingulum was associated with a decline in episodic visual memory (27). We also recorded highly significant associations between right ventral cingulum with worse episodic visual memory (increased MD and reduced FA) together with deficits in visuospatial ability and executive function. Worse performance in executive function has been previously shown in CPs (7, 9), but for the first time, we find an association to a specific WM tract.

There are scarce data involving DTI in the field of endocrinology (18, 23, 39, 40, 41, 42), and recent studies on cognitive function have focused on brain volumes and cortical thickness (18, 39, 40). Gray matter area is involved in episodic memory and includes the cingulate cortex situated above the cingulum, and the posterior cingulate cortex is linked to the hippocampus via its abundant connections with the cingulum. Özyurt *et al*. (18) introduced results supporting the theory that HL impacts gray and white matter outside the area of CP tumor growth. By analyzing gray matter volume in the posterior cingulate cortex, they recorded a positive association with episodic memory (18). Among patients with Cushing syndrome widespread WM involvement on DTI was recorded with both a decrease WM integrity (decrease in FA) and demyelination/edema (increase in MD) (41). Their data were predominant for demyelination and seemed to be independent of concomitant hypercortisolism and cardiovascular risk factors (41).

It is well known that the hippocampal volume decreases in size with increasing age, even in healthy adults (43). Thus, we adjust not only for age but also for ICV. The CP patients had lower right and left hippocampal volumes but among patients the variation in ICV explains 34% of the variation in total hippocampal volume. Thus, patients tend to have a smaller ICV, including the hippocampus, which may be treatment related. In other words, the treatment effect may be a generalized decrease in brain volume rather than an isolated effect on hippocampal volumes. The reduced hippocampal volumes were associated with lower performance in general knowledge and in episodic visual memory. The latter is in accordance with a study involving a group of patients with Cushing syndrome with reduced hippocampal volume who suffered from impaired episodic visual memory (39). Regarding DTI measures in the hippocampus, we adjusted only for age as we assumed that ICV did not serve as a confounder but was more part of a chain of disease events. We recorded alterations in WM integrity (decrease in FA) in the right hippocampus, which was associated with reduced visuospatial ability, and the left hippocampus was altered by both loss of WM integrity and demyelination/edema, which was associated with worse performance in general knowledge and in episodic visual memory for delayed recall. Thus, based on the present alterations in hippocampus, there were many potential reasons for a lower performance in cognition.

This is the first study presenting DTI in CP with HL, which also was reflected in a reduction in the number of patients included for the overall DTI analyses (*n* = 15), resulting in only 8 patients with HL and sufficient DTI quality. Thus, our nonsignificant results could be due to low statistical power. Data on CP patients with intact HT are published elsewhere, showing no differences in DTI measures between patients and controls (23). These results are in line with the fact that this subgroup of CP patients, with intact HT, has a much better prognosis and cognitive function (7).

Our study has a number of limitations. Cognitive function depends on complex neural networks, and it is impossible to relate a certain memory function to a single specific neural pathway. Further, the study design does not allow for any assumptions regarding causality nor the pathogenesis behind the WM alterations, as e.g. cranial radiotherapy was closely related to extensive re-operations and to HL. Also the necessary hormone replacement, might to some point have an impact on the present result. The HT damage caused by the tumor or its treatment could be the direct cause of the WM alterations associated with the recorded cognitive deficits, but, also indirectly, as the connectivity between several HT compartments and the neural tracts (15) has been disrupted due to the tumor and/or treatment. Due to the rarity of the disease, the study population is small. Nevertheless, with a background population of 2.5 million, we included the eligible surviving adult population of childhood-onset CP, during 52 years in this area of Sweden. We chose to compare our results to DTI measures in Alzheimer disease as it is most relevant for episodic memory. Another factor important to keep in mind when comparing studies is the heterogeneity in as to how different segments of the cingulum are defined (44).

## Conclusion

For the first time, a structure to function relationship is established between WM alterations detected with DTI and cognitive dysfunction in CP. The strongest associations were found between WM alterations in the cingulum, with both loss of WM integrity and demyelination/edema, to a decline in visual episodic memory. Further, CP patients have a smaller hippocampus, but also reduced WM integrity and demyelination, associated with lower performance in general knowledge. New knowledge and increased awareness of cognitive dysfunction is essential for optimizing postoperative outcome and care among CP patients with HT damage.

## Supplementary Material

Supporting Table 1

## Declaration of interest

The authors declare that there is no conflict of interest that could be perceived as prejudicing the impartiality of this study.

## Funding

This work was supported by the Swedish Children’s Cancer Foundation, and the Medical Faculty, Lund University, Sweden.

## Author contribution statement

All authors helped to conceptualize the study. S B F, E M E, L R conducted the data analysis. D S and D V W analyzed the tractography. All authors helped to interpret the data. S B F drafted the original paper and EME extensively revised the paper with input from all authors. All authors approved the final version.
