# Supplementary material for: Microstructural white matter alterations and hippocampal volumes are associated with cognitive deficits in craniopharyngioma
Source: Eur J Endocrinol. 2018 Mar 16;178(6):577–87. doi: 10.1530/EJE-18-0081 (PMC5937918; doi:10.1530/EJE-18-0081)
Supplement: Supporting Table 1 [file eje-178-577-t001.pdf]

**Supplemental Table 1.** Description of vision and visual fields in 39* patients with childhood onset craniopharyngioma who performed cognitive testing.

| | With Hypothalamic lesion | Without Hypothalamic lesion | Total |
|---|---|---|---|
| Substantial visual field defects **and** vision ≤ 0.5 on both eyes | 2 | 2 | 4 |
| Substantial visual field defects **and** vision ≤ 0.5 on one eye, normal vision other eye | 7 | 1 | 8 |
| Substantial visual field defects **and** normal vision | 1 | 1 | 2 |
| Minor visual field defects **<u>or</u>** normal visual fields **and** normal vision | 12 | 13 | 25 |
| Total | 22 | 17 | 39 |

* The remaining 2 patients who performed cognitive testing were hard to categorize and were as follows:

1. Unilateral hemianopsia with vision <0.5 on one eye, normal vision other eye (intact hypothalamus)
2. Normal visual fields with <0.5 on one eye, normal vision other eye (hypothalamic lesion)

Substantial visual field defects: Decreased vision or blindness in at least one quarter of the visual field bilaterally
